# Empyema thoracis: A clinical study

**DOI:** 10.4103/1817-1737.30356

**Published:** 2007

**Authors:** Preetam Rajgopal Acharya, Kusum V. Shah

**Affiliations:** Dept. of TB and Chest Diseases, Kasturba Medical College, Attavar, Mangalore, Karnataka, India; * *Dept. of TB and Chest Diseases, B.J. Medical College, New Civil Hospital, Meghaninagar, Ahmedabad, India*

**Keywords:** Closed tube thoracostomy, empyema, parapneumonic effusion

## Abstract

**BACKGROUND:**

Empyema thoracis is a disease that, despite centuries of study, still causes significant morbidity and mortality.

**AIM:**

The present study was undertaken to study the age-sex profile, symptomatology, microbiologic findings, etiology and the management and treatment outcome in a tertiary care hospital.

**SETTINGS AND DESIGN:**

A prospective study of empyema thoracis was conducted on 40 consecutive patients with empyema thoracis admitted to the tuberculosis and chest diseases ward of a teaching hospital.

**MATERIALS AND METHODS:**

The demographic data, clinical presentation, microbiological findings, etiology, the clinical course and management were recorded as per a planned pro forma and analyzed.

**RESULTS:**

The peak age was in the range of 21-40 years, the male-to-female ratio was 3.4:1.0 and the left pleura was more commonly affected than the right pleura. Risk factors include pulmonary tuberculosis, chronic obstructive pulmonary diseases, smoking, diabetes mellitus and pneumonia. Etiology of empyema was tubercular in 65% cases and nontubercular in 35% cases. Gram-negative organisms were cultured in 11 cases (27.5%). Two patients received antibiotics with repeated thoracentesis only, intercostal chest tube drainage was required in 38 cases (95%) and more aggressive surgery was performed on 2 patients. The average duration for which the chest tube was kept in the complete expansion cases was 22.3 days.

**CONCLUSION:**

It was concluded that all cases of simple empyema with thin pus and only those cases of simple empyema with thick pus where size of empyema is small should be managed by aspiration/s. Cases failed by the above method, all cases of simple empyema with thick pus and with moderate to large size of empyema and all cases of empyema with bronchopleural fistula should be managed by intercostal drainage tube connected to water seal. It was also observed that all cases of empyema complicated by bronchopleural fistula were difficult to manage and needed major surgery.

An empyema is, by definition, pus in the pleural cavity. The diagnosis and treatment of empyema by surgical drainage was first described by Hippocrates in around 600 B.C.[1] Since then the management of this condition has posed a challenge to physicians and surgeons alike. From ancient times to the middle of last century, most empyemas were the result of pneumonic, traumatic or tuberculous processes. With thoracotomy becoming a commonly performed procedure, postsurgical empyema now constitutes 20% of all cases.

The empyema commission formed to address the high mortality rates secondary to the institution of open drainage in all cases of empyema occurring in American soldiers during the World War I laid emphasis on the following principles: 1. the necessity to drain the pleural fluid and the need to avoid an open pneumothorax in the acute pneumonic phase, 2. the rapid sterilization and obliteration of the infected cavity in order to avoid a chronic empyema and 3. proper nutrition of the patient. The same guidelines framed decades ago continue to provide the basis for the treatment of empyemas even today.[2]

Tube thoracostomy, image directed catheters, thoracoscopic drainage, intrapleural thrombolytics, decortications and open drainage have all been used with success rates ranging from 10 to 90%.[3,4] The variable success rates of these procedures can be attributed, in part, to the stage of the empyema at presentation.

The present study is aimed at studying the etiology, bacteriological features, the clinical presentation, diagnostic modalities, the different modalities of treatment available for this condition and the overall treatment outcome in a teaching hospital.

## Materials and Methods

This is a cross-sectional study of 40 consecutive indoor patients with empyema thoracis admitted to the tuberculosis (TB) and chest diseases ward at New Civil Hospital, Ahmedabad, India, conducted between March 1998 and November 2000. Posttraumatic and postsurgical empyema patients were excluded from the study. Diagnosis was established by the aspiration of gross pus from the pleural space. Cases wherein the aspirated pleural fluid was nonpurulent, a positive gram stain or a positive culture was also accepted as empyema and included in the study. A detailed history was taken in all cases as per a planned pro forma. Besides the routine investigations, pleural fluid was sent in all cases for gram stain and Ziehl Neelsen stain (ZN), and culture sensitivity was done for both aerobic pyogenic organisms and mycobacteria (Lowenstein Jensen method). Sputum was examined for acid fast bacilli (AFB) in all cases. Radiological assessment included chest radiographs taken at admission, after intercostal tube insertion and after ICD removal/at discharge. Where needed, ultrasound thorax or computed tomography (CT) of the thorax was done. Evaluation of these patients included the demographic profile, the presenting clinical features, the organisms isolated, etiology of the empyema and the response to various modes of treatment. The data was analyzed using the method of descriptive statistics and the results expressed as percentage of the total.

## Results

A total of 40 patients were included in the study – male: 31 (77.5%), female: 9 (22.5%). Thus the male-to-female ratio was 3.4:1.0. Their mean age was 33.12 years. Majority of the patients, i.e., 42%, were in the age group 21–40 years. Twenty-two patients (55%) were residents of urban areas and 18 patients (45%) belonged to rural areas.

The commonest symptoms at presentation were cough, seen in 37 patients (92.5%); dyspnea (37, 92.5%); followed by fever (35, 87.5%) and chest pain (32, 80%). In addition, constitutional symptoms, viz., anorexia, malaise and weight loss, were noted in 25 patients (62.5%) [Table 1].

**Table 1:** Clinical presentation of empyema thoracis

Symptoms	Present study n (%)
Cough	37 (92.5)
Dyspnoea	37 (92.5)
Chest pain	32 (80.0)
Fever	35 (87.5)
Constitutional symptoms	25 (62.5)

Past history of tuberculosis was available in 18 patients (36%), and 4 patients (10%) had history of chronic obstructive pulmonary diseases (COPD). In the present series, 3 patients (7.5%) were diabetics, and 22 patients (55%) were smokers.

In the present series, gram-negative bacilli were isolated most frequently from empyema fluid in 11 patients (27.5%), while mycobacteria was cultured in 6 (15%) and staphylococcus in 5 (12.5%). In 22 patients (55%), the pus was sterile [Table 2].

**Table 2:** Empyema thoracis: Bacteriological profile

Organism	Present study (n)	Present study (%)
Staphylococcus	5	12.5
Pneumococcus	0	0
Streptococcus	1	2.5
Gram negative bacilli	1	27.5
Mixed pyogenic	3	7.5
Sterile	22	55
Mycobacteria		
1. Pure	6	15
2. Mixed: AFB plus	3	7.5
Other bacilli.	3	7.5

Sputum was positive for AFB in 10 patients (25%) of the current series. AFB was identified in the empyema fluid by ZN stain in 12 patients (30%). Culture for AFB was positive in 6 patients (15%).

Radiologically, 8 patients (20%) had a ‘meniscus sign,’ suggesting effusion, whereas 30 patients had an air-fluid level suggestive of hydropneumothorax. Two patients (5.0%) presented with radiological features of encystement.

Of the 26 patients diagnosed as having tubercular empyema, the diagnosis was established bacteriologically, i.e., based on the presence of AFB in sputum and/or empyema fluid in 19 patients (73%). In the remaining 7 cases (27%), this diagnosis was based on clinical grounds, i.e., previous h/o pulmonary tuberculosis, presence of tuberculous toxemia, radiological appearances consistent with active tuberculosis and response to anti-tuberculous treatment [Table 3].

**Table 3:** Tubercular empyema

Diagnostic criteria		Present study (n)	Persent study (%)
1.	Based on bacteriological criteria: Detection of AFB in sputum and/or pus.	19	73
2.	Based on clinical and radiological criteria: Presence of tuberculous toxaemia and/or previous h/o pulmonary tuberculosis and/or chest X-ray suggestive of T.B and/or therapeutic response to anti-tuberculous treatment but sputum and pleural pus negative for AFB.	7	27
	Total	26	100.0

AFB - Acid fast bacilli

Only in 2 patients of the present study, the condition resolved with antibiotics and repeated therapeutic aspirations. Closed drainage (tube thoracostomy connected to an underwater seal drainage) with use of systemic antibiotics was the therapeutic approach used most commonly in the series: 38 patients (95%). Two patients were referred to the cardiovascular thoracic surgery department for decortication. Both patients had chronic empyema with bronchopleural fistula (BPF).

Out of 40 patients, 25 (62.5%) had complete expansion of the lung, while 12 patients (30%) had only partial expansion [Table 4]. Patients with partial expansion mainly comprised of those who presented late in the course of the disease, i.e., chronic empyema with thickened pleura, and those who developed a chronic BPF after ICDT. The average duration for which the ICD was kept in the complete expansion cases was 22.3 days. The commonest complication observed following chest tube insertion was surgical emphysema.

**Table 4:** Treatment outcome

Outcome	Present study (n)	Present study (%)
Complete expansion	25	62.5
Partial expansion	12	30.0
Left against medical advice	1	2.5
Transfer to cardiothoracic surgeon	2	5.0
Expired	0	0.0
Total	40	100.0

## Discussion

The high incidence of empyema in the productive age group of 21-40 years in this study is consistent with the findings in the earlier study by Behra and Tandon.[5,6] This may be due to the common occurrence of pulmonary tuberculosis in this age group, particularly in the developing countries with a high prevalence of tuberculosis. Two other studies show the incidence of empyema to be higher after the age of 40.[7,8] This may be attributed to the fact that the above studies were carried out in the developed countries, where the overall prevalence of tuberculosis is relatively low; in contrast to the present study, which was undertaken in India.

In the present study, males outnumbered female patients in the ratio of 3.4:1.0. Males in general are more prone to mechanical stresses due to their tall stature and strenuous work. Smoking is a more frequent habit, and tuberculosis and COPD are more frequent in males.

The study done by Kamat reported cough (94%) to be the most common symptom. This was followed by fever (76%), chest pain (75%) and dyspnea (53%).[9] The prevalence of cough (92.5%), chest pain (80%) matches that of the study by Kamat, whereas dyspnea (92.5%), fever (87.5%) and constitutional symptoms (62.5%) were encountered more frequently in our patients. The clinical manifestations of an empyema can vary widely, depending on both the nature of the infecting organism and the competence of the patient's immune system. The spectrum ranges from an almost complete absence of symptoms to a severe illness with systemic toxicity.[10] In general, anaerobic and tubercular empyemas usually present with a subacute illness, whereas aerobic bacterial infections of the pleural space present with an acute illness.

In the present study, infectious etiology is more common because this study was carried out in a TB and chest diseases ward and patients of posttraumatic and postsurgical empyema were excluded from the study. In 26 patients (65%) the empyema was tubercular in etiology. In 12 patients (30%) the empyema developed as a complication of bacterial pneumonia, and in 2 patients (5%) it was secondary to a lung abscess.

Prior to the availability of antibiotics, streptococcus pneumoniae and streptococcus pyogenes accounted for most empyemas. After the discovery and widespread use of penicillin in the 1940s, staphylococcus aureus succeeded *S. pneumoniae* and *S. pyogenes* as the major cause of empyema. Since the advent of β-lactamase resistant semisynthetic penicillins in the early 1960s, the incidence of staphylococcal empyema has decreased, and infections due to anaerobic bacteria (Bacteroides, Peptostreptococci and Fusobacteria) and aerobic gram-negative bacilli (*E. coli,* Pseudomonas, Proteus, Klebsiella) have increased markedly. Approximately 75% of patients with empyema have multiple infecting organisms, averaging three bacterial species per patient. The pus is found to be sterile in only one-third of cases.[11] The pathogen isolated in empyema also depends on presence or absence of certain predisposing factors like community-acquired pneumonia (*S. pneumoniae*), h/o aspiration (anaerobes), subdiaphragmatic infections (aerobic gram-negative enteric bacilli), external trauma and hemothorax (*Staphylococcus aureus*) and immunosuppression (*Staphyhlococcus aureus, Mycobacteria, fungi*). Age is also an important deciding factor. Whereas coagulase-positive *Staphylococcus aureus* is common in childhood, gram-negative organisms other than Hemophilus influenza are relatively unusual causes of empyema in children; anaerobic organisms are rare in patients younger than 18 years.[10] In the present study, a positive culture was obtained in 18 patients (45%). Gram-negative organisms were cultured most frequently (27.5%). This is in concurrence with the reports of various workers who have emphasized the emergence of gram-negative bacilli as predominant pathogen.[7,8] In our series pleural fluid was sterile in 55% cases. This high negative culture report, compared to the previous studies, correlated with the high number of tuberculous empyema thoracis in the present study.

A good proportion of cases of tubercular empyema can be diagnosed by isolation of AFB in sputum and/or pleural pus. In addition, a smaller number were diagnosed on basis of past history, symptomatology, radiological lesions and therapeutic response to anti-tuberculous treatment [Table 3].

There are two basic principles for the successful management of thoracic empyema – namely, the control of infection with appropriate antimicrobial therapy and the adequate drainage of pus.

The initial choice of antibiotics depends on the results of Gram's stain of pleural fluid and sputum. This is then modified as per the clinical response of the patient and the result of culture and sensitivity. As a rule, antibiotics are continued until (1) the patient is afebrile and the white blood cell count is normal, (2) the tube thoracostomy drainage yields <50 ml of fluid daily, (3) the radiograph shows considerable clearing. Typically, antibiotics are required for 3 to 6 weeks.[11]

The choice of the method used to drain the empyema depends on the stage of the empyema. The pleural fluid in the first stage, i.e.,*the exudative stage,* is small in volume, sterile and free flowing. It is characterized by a low white blood cell count and lactic acid dehydrogenase (LDH) and a normal glucose level and pH. An empyema detected at such an early stage can be managed by antibiotics plus serial thoracentesis.[10,12]

The second stage, viz., *the fibrinopurulent stage,* is characterized by an increase in the volume of pleural fluid, which is then turbid in appearance due to the presence of a large number of polymorphonuclear leukocytes, bacteria, fibrin and cellular debris. The pleural fluid pH and glucose progressively decline and the LDH level progressively increases. Septations form in the pleural cavity to contain the infection and prevent the extension of the empyema.[10,12] Management of this stage of empyema necessitates the use of chest tubes. Successful closed-tube drainage is evidenced by improvement in the clinical and radiologic status within 48 h. If the patient fails to improve clinically and radiographically within 48 h, ultrasonic or CT examination of the pleural space is performed to detect undrained loculated fluid. In patients with inadequate drainage, the choices are 1. image (CT/ultrasound) guided placement of additional chest tubes; 2. suction therapy, i.e., the use of negative suction to break loculations; 3. use of intrapleural fibrinolytic agents like streptokinase and urokinase; 4. video-assisted thoracoscopic surgery with breakdown of adhesions; 5. thoracotomy with digital lysis of adhesions and operative placement of chest tubes with or without decortication.[11]

The formation of a fibrinous inelastic membrane on the surface of both the pleura – variously referred to in literature as the pleural cortex, the rind, peel – encases the lung, rendering it functionless; these are features of the third stage of empyema, or *the organizational stage*.[10,13] This stage of empyema requires surgical intervention, which usually takes the form of decortication. Decortication is an elective surgical procedure in which the fibrous wall of the empyema cavity – the cortex, rind or peel – is stripped of the adjacent visceral and parietal pleura along with the evacuation of pus, blood clots and fibrin material from pleural cavity. This eliminates the pleural sepsis and allows the underlying lung to expand.[2,10,13]

With the higher percentage of use of chest tubes in the present study (95%), it could be postulated that we are more successful in the management of this process with closed tube thoracostomy only. A large number of cases in the present study were unsuitable for surgical therapy because of involvement of both or contralateral lung with tuberculous pathology. Delay in treatment increases morbidity, and decortication is more effective than open drainage in reducing morbidity and mortality when surgical intervention is necessary.[12]

The mortality from empyema ranges from 11 to 50%. A good outcome demands prompt recognition, appropriate antibiotic therapy and adequate pleural drainage. Pleural fluid of pH <7.2, glucose <40 mg/dl and LDH >1,000 IU/L indicates a patient with increased risk of needing pleural drainage.[11] However, in practice such criterion may not always be readily available to guide treatment decisions. Hence we propose that in absence of the above, size of the effusion and thickness of aspirated fluid may be used to guide the need for a chest tube placement. Cases failed by repeated thoracentesis, all cases of simple empyema with thick pus and with moderate to large size of empyema and all cases of empyema with bronchopleural fistula (BPF) should be managed by intercostal drainage tube connected to water seal.
